# ICD-11: an international classification of diseases for the twenty-first century

**DOI:** 10.1186/s12911-021-01534-6

**Published:** 2021-11-09

**Authors:** James E. Harrison, Stefanie Weber, Robert Jakob, Christopher G. Chute

**Affiliations:** 1 College of Medicine and Public Health, Flinders University, Adelaide, Australia; 2 Federal Institute for Drugs and Medical Devices, Bonn, Germany; 3 World Health Organization, Geneva, Switzerland; 4 Schools of Medicine, Public Health and Nursing, JohnsHopkins University, Baltimore, MD USA

**Keywords:** Epidemiology, Informatics, Statistics, eHealth, Classification, International classification of diseases

## Abstract

**Background:**

The International Classification of Diseases (ICD) has long been the main basis for comparability of statistics on causes of mortality and morbidity between places and over time. This paper provides an overview of the recently completed 11th revision of the ICD, focusing on the main innovations and their implications.

**Main text:**

Changes in content reflect knowledge and perspectives on diseases and their causes that have emerged since ICD-10 was developed about 30 years ago. Changes in design and structure reflect the arrival of the networked digital era, for which ICD-11 has been prepared. ICD-11’s information framework comprises a semantic knowledge base (the Foundation), a biomedical ontology linked to the Foundation and classifications derived from the Foundation. ICD-11 for Mortality and Morbidity Statistics (ICD-11-MMS) is the primary derived classification and the main successor to ICD-10. Innovations enabled by the new architecture include an online coding tool (replacing the index and providing additional functions), an application program interface to enable remote access to ICD-11 content and services, enhanced capability to capture and combine clinically relevant characteristics of cases and integrated support for multiple languages.

**Conclusions:**

ICD-11 was adopted by the World Health Assembly in May 2019. Transition to implementation is in progress. ICD-11 can be accessed at icd.who.int.

## Background

Understanding diseases in ways that enable prevention, treatment, and the allocation of resources requires measurement. To be useful, measurement must be reliable, allow valid comparisons to be made between places and over time, and enable coherent summarization of large volumes of data. A classification of diseases and related things is essential for such measurement.

For more than a century, the International Classification of Diseases (ICD) has been the main basis for comparable statistics on causes of death and non-fatal disease [1, 2]. The 10th revision (ICD-10) was released nearly 30 years ago [3]. It serves a variety of functions in much of the world—at least 120 countries—and has been translated into 43 languages [4].

Uses of the ICD are diverse and widespread, extending directly to much of the world and indirectly to all populated places. Much of what is known about the extent, causes and consequences of human disease world-wide rests on use of data classified according to the ICD. Clinical modifications of ICD are the main basis for statistics on disease, particularly cases treated by hospitals. These statistics underlie crucial functions such as payment systems, service planning, administration of quality and safety and health services research.

This essential infrastructure for health information has now been revised for the 11th time. The 11th revision was more extensive and has greater implications for what can be done with the ICD, and how, than any revision since the 6th, in 1948.

Since the development of ICD-10, medicine has advanced, and the understanding of many diseases has changed substantially. The modifications needed to accommodate these changes exceeded what could be achieved by simply updating the 10th revision. Another reason for undertaking a major revision of the ICD is an extrinsic factor, which now affects almost all areas of life: the arrival of the digital age [5]. While ICD-10 has long been used in digital forms, properties that reflect its pre-digital origin constrain tooling and data exchange, impede maintenance and development, and have enabled differences of structure and meaning to creep into translations and modifications.

The 11th revision [6], adopted by the 72nd World Health Assembly in May 2019 after extensive consultation and deliberation [7–21], addresses these shortcomings of ICD-10 and more. In aggregate, the changes are substantial: ICD-11 is not just ICD-10 with some new categories. Rather, ICD-11 is a different and more powerful health information system, based on formal ontology, designed to be implemented in modern information technology infrastructures, and flexible enough for future modification and use with other classifications and terminologies. It is better able to capture clinically relevant characteristics of cases and to permit summarization of information for various purposes, has flexibility allowing use in more and less elaborate modes, and has integrated support for multiple languages. It is also designed to ensure that data coded according to ICD-11 will be comparable with data coded to ICD-10.

This commentary introduces the conceptual basis for the design of ICD-11 and provides an overview of the content and the most important features.

## Main text

### The design of ICD-11

#### Information framework

Fundamental to making the ICD fit for the digital age has been to base it on a computable knowledge framework. This is the most important difference between ICD-11 and earlier revisions. Introduction of a knowledge framework has enabled ICD-11 to be interoperable in digital health information environments. Though ICD-11 can be used in paper-based systems, the tools and capabilities made possible by the framework are expected to make electronic use compelling for most users. The first derived classification, ICD-11 for Mortality and Morbidity Statistics (ICD-11-MMS), is the most direct successor to ICD-10.

The information framework for ICD-11 has three integrated parts: a database referred to as the Foundation, classifications derived from the Foundation, and a common biomedical ontology linked to the Foundation. These components are described below.

#### The Foundation

The Foundation has about 80,000 entries complemented by 40,000 synonyms, each characterizing a disease, syndrome, or health-related phenomenon in a way that not only is descriptive but also specifies its relationships with other entities and provides a way for digital systems to take account of meaning that may be assigned to an entity. In other words, the Foundation is a semantic network.

A template, or content model [22], specifies what must be or can be recorded in each entry. Attributes that are well populated on release include a unique, unchanging identifier, preferred name, fully specified name, synonyms, human language translations of names and synonyms, a description (approximating a definition), notes, details of severity grades or stages, parent relationships, and child relationships. The template also includes elements that are more aspirational, such as genomic associations, etiology, clinical criteria and manifestations.

Statistical classifications, such as the ICD-11-MMS, have the property of mutual exclusivity: each codable concept must be located in only one place in the classification’s hierarchy. Many concepts in the ICD have properties that relate them to more than one part of the hierarchy. For example, stroke involves the circulatory system and results in neurological disease. Provision of suitable categories for stroke is more important than where they are placed, though placement is a matter on which strong views sometimes exist [23, 24]. The Foundation reduces the impact of such choices by allowing a concept to have many parents.

This property of the Foundation is useful in classifications based on it. For example, while stroke is located in the neurology chapter of ICD-11-MMS, the disease also appears in the cardiovascular chapter (where it was located in ICD-10), with an indication that its primary location is elsewhere. Hence, multiple parenting in the Foundation allows categories to appear in ICD-11 classifications where various users might expect to find them.

Practical considerations dictate that a statistical classification will comprise a limited number of categories, constraining the scope and specificity of the phenomena that can be coded in ICD-11-MMS. The Foundation is practically unlimited in this respect. Specificity implicit in the Foundation that goes beyond that in ICD-11-MMS can be used by other classifications, which are expected to be derived from the Foundation.

Primary authoring of ICD-11 was done in English. However, each preferred term, fully specified term, and synonym is being rendered into the other standard languages of the World Health Organization (WHO)—Arabic, French, Mandarin, Russian, and Spanish—by means of a large multilingual phrase thesaurus, accumulated from translations of previous revisions of the ICD, with trained translators checking the results. Some other languages are also included, and the number will increase as ICD-11 comes into use.

#### Linearization

As a statistical classification, the ICD-11-MMS has special properties, notably mutual exclusivity of categories, exhaustive coverage of the domain of interest, and arrangement as a single hierarchical tree. In contrast, the Foundation embodies a rich network of relationships between entities, in which there is no constraint on entities having more than one logical parent. That is, many hierarchies are implicit in the Foundation. Deriving this particular statistical classification from the Foundation required use of a process known in information science as linearization [25].

**Fig. 1:**
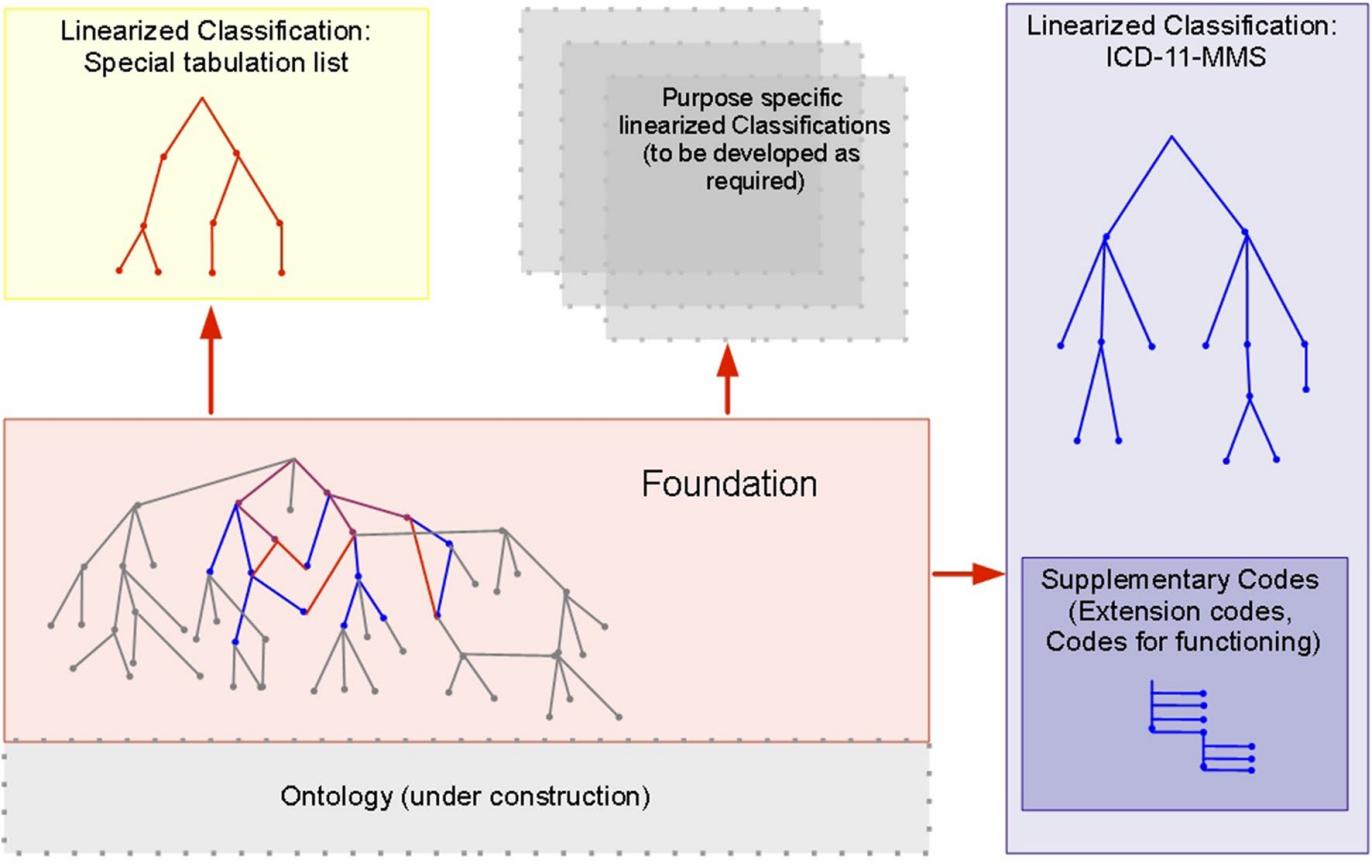
The ICD-11 Foundation and classifications based on it, including ICD-11 for mortality and morbidity statistics (ICD-11-MMS). Many classifications can be linearized from the rich Foundation. ICD-11-MMS (the main classification; blue) and the special tabulation list (a short set of categories for standard summary reports; red) share some concepts in the Foundation (purple). Concepts in the Foundation that are not included in ICD-11-MMS classification (grey) are, nevertheless, part of its index. Practicalities for use of ICD-11-MMS required that it should be constrained to a modest number of codable categories. Great extension of the expressive capabilities of ICD-11-MMS is provided by permitting code clusters to be built by combining stem codes and adding supplementary codes, chiefly extension codes (see Table 1)

Linearizing the ICD-11-MMS from the Foundation involved deciding which entities in the Foundation would be included in the classification, deciding on the depth of its hierarchy, and putting each selected entity into a single place in the classification hierarchy. Other classifications can also be linearized from the Foundation (Fig. 1). Decisions on which entities to include in each and how to organize the hierarchy can be expected to differ according to the purpose in mind (e.g., a clinical specialty such as dermatology, primary care, or public health). The family of classifications linearized from the Foundation has the special property that mapping or cross-walking between terms can be done reliably.

As is typical of statistical classifications, achieving the property of exhaustiveness for ICD-11-MMS required the addition of residual categories (“other specified,” “unspecified”). Residuals are not part of the Foundation and are only meaningful in the context of a particular classification.

#### Common ontology

The Foundation is a semantic knowledge base. The entities used in the knowledge base, and how they are represented, are referred to by the information science term 'ontology' [25]. The Foundation can be anchored to external ontology sources by inclusion and reconciliation of terms and definitions, with attribution. Some preliminary demonstrations [26, 27] were done with SNOMED [28], with others planned for the Human Phenotype Ontology [29] and MedDRA [30]. Future options may include other members of the Open Biomedical Terminologies (OBO) [31] community.

The more adequately the ontology underlying ICD-11 represents the relevant domain of knowledge the more straightforward it should be to incorporate new entities. For example, the SARS-CoV-2 virus, the COVID-19 disease, and various manifestations of the disease, which emerged after the release of ICD-11, were easily incorporated as new instances of relevant dimensions of the ICD-11 content model.

### Clustering

In ICD-10 a diagnosis is usually represented by a single code. A limited exception was provided by the dagger-asterisk convention, which allowed a code representing the etiology of a disease (e.g., diabetes) to be linked with a code representing a manifestation (e.g., retinopathy). Likewise, the external causes of injuries are commonly coded as well as their diagnoses. ICD-10 lacks a well-developed infrastructure to support linking of codes, and information for this is often not captured or is lost in processing.

**Fig. 2:**
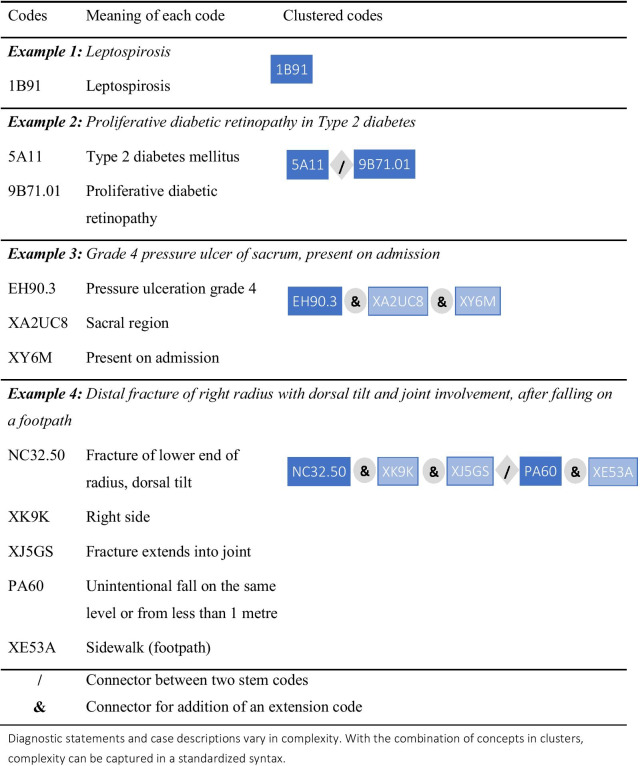
Examples of ICD-11-MMS code clusters

ICD-11-MMS provides a general mechanism to allow codes to be combined to form clusters for use where expressive power is required beyond that provided by any single category (Fig. 2). In principle, any ICD-11-MMS category that can be coded on its own (a “stem code”) can also be clustered with one or more other stem codes. A stem code can also be qualified by being clustered with one or more “extension codes”, which can only be used in clusters (Table 1). ICD-11-MMS provides over 20,000 extension codes, many of which are of a few types, such as a hierarchical list of drugs and other substances that might cause poisoning, or harm health in other ways. These extension codes allow further specificity and additional information to be added to stem codes.

**Table 1:** The topics of extension codes in ICD-11-MMS, with examples

Topics	Types and examples
Severity scale value	None, mild, moderate…. Grade 0/1/2…
Temporality	Course of the condition. Time in life. Pregnancy duration
Aetiology	Causation. Infectious agents. Allergens
Topology scale value	Relational. Distributional. Laterality. Regional
Anatomy and topography	Functional anatomy. Body regions. Partonomic view
Histopathology	Gliomas: benign, malignant, uncertain, in situ
Dimensions of injury	Types of fractures and whether open or into a joint
Dimensions of external causes	Type and part of place. Activity. Objects. Alcohol and drugs
Consciousness	Components of Glasgow Coma Scale. Pupil reaction
Substances	Medicaments (e.g., oxycodone). Chemicals (e.g., parathion)
Diagnosis code descriptors	Diagnosis timing/certainty/method of confirmation
Capacity or context	Condition of fetus/newborn reported in context of the mother
Health devices, equipment, and supplies	Medical devices, surgical instruments, dialysis supplies

Users and their purposes will determine how much detail is necessary, and ICD-11-MMS provides great flexibility in this regard. While extensive clustering will be necessary to meet the needs of some users, others might require little or none, and ICD-11-MMS has been designed to provide coherent information and the basis for useful statistical comparisons if only a single stem code is assigned to a main diagnosis or underlying cause of death.

Quality and safety of health care exemplifies the potential of clustering. Consider a person admitted to hospital for a surgical procedure who experiences a complication of care. ICD-11-MMS allows for coding of the disease for which surgery was undertaken (that would be the subject of one cluster) and of the complication. A code cluster on the latter can record the harm sustained (e.g., marked nausea and vomiting after surgery), the medication involved (perhaps a particular anesthetic agent), and how the problem came about (e.g., dose too high or too low, or administered at the wrong time). Extension codes can also record whether a condition had been recognized as present when the episode of care began.

#### Support for digital communication

Every distinct concept in the ICD-11 has been assigned a unique and unchanging identifier. This unique resource identifier (URI) remains the same whether the concept appears solely in the Foundation or is also included in the ICD-11-MMS (or another classification) linearized from the Foundation. The URIs are for use by digital systems and are intentionally “meaningless identifiers” [32], which enable many of the capabilities of ICD-11. For example, they will enable a health information system to reliably locate information on a topic in any of the languages available on the WHO platform, facilitating health care across borders. The URIs are distinct from ICD-11-MMS codes, which are not arbitrary, reflect aspects of the classification tree, and are for use by humans.

ICD-11 is a unique knowledge base of diagnostic concepts and related matters. The URIs identify the numerous entities within it but do not, on their own, provide users with a way to tap its potential. To enable that, the WHO has created a suite of application programming interfaces (APIs), or RESTful web resources [33]. These will allow developers anywhere to integrate access to ICD-11 and its services into software, such as coding support applications, and to use the URI to retain the exact detail of a term in addition to the statistical code or code combinations.

#### Coding tool as index

Use of the index volume is the recommended way to find the appropriate code for a disease in ICD-10 and earlier revisions [34]. ICD-11 provides users with a different way to find and select categories. The digital capabilities described above have been used by the WHO to create a web-based coding tool [35] that employs partial word-matching, word-order independence, synonym management, hierarchy traversal, and more. Where a search term equates to a cluster, rather than to a single stem code, the tool can return the assembled cluster.

Application prototypes using the ICD-11 API have been demonstrated for mobile devices, making electronic access to ICD coding available just about anywhere. Low-resource areas and sites will be able to use these free resources with inexpensive devices, which are likely to be less costly and more portable than bulky printed volumes and indices. Field testing of the coding tool and system show promising indications that it will result in more comparable, consistent, and accurate coding than did the previous approach.

### Current medical knowledge

The need to accommodate new knowledge about diseases and changes in related concepts and terms necessitated many changes in the ICD. In fact, the need for updates was noted soon after ICD-10 was published. An updating mechanism was put into place, but structural characteristics of ICD-10 and requirements for continuity limited the types of changes that could be made, and a growing list of desired changes were put aside for the 11th revision.

A design objective for the 11th revision was to maintain good backwards comparability with ICD-10, particularly for important conditions, and changes were made only where credible reasons emerged to do so. Despite the many changes at specific levels, the overall framing of diseases in ICD-11-MMS remains similar to that in ICD-10, and this is reflected in the similar titles and sequence of chapters. Several new chapters and sections have been added for various reasons (Table 2). Specific changes include the addition of categories for new concepts, splitting and lumping of old categories, retirement of redundant categories, rewording of titles, and movement of categories from one place to another within the classification tree.

**Table 2:** New chapters and sections in ICD-11-MMS

Title	Reason for addition
Chapter 3: Diseases of the blood or blood-forming organs Chapter 4: Diseases of the immune system	These two chapters were split from a single chapter in ICD-10, recognizing differences in etiology, manifestations, and care
Chapter 7: Sleep–wake disorders	This topic has become more prominent since the 10th revision. The chapter mostly includes new concepts with some concepts moved from other chapters in ICD-10
Chapter 17: Conditions related to sexual health	This topic has become more prominent since the 10th revision. The chapter mostly includes concepts moved from other chapters in ICD-10, combined with some new concepts
Chapter 26: Traditional medicine conditions	This entirely new supplementary chapter in ICD-11 enables coding in terms of traditional medicine concepts, where required
Extension codes section	Codes in this section can be combined with a stem code to provide additional information
Functioning section	Some national modifications of ICD-10 added sections to allow patient functioning to be recorded. ICD-11 provides a supplementary section for functioning assessment, aligned with the WHO International Classification of Functioning

**Fig. 3:**
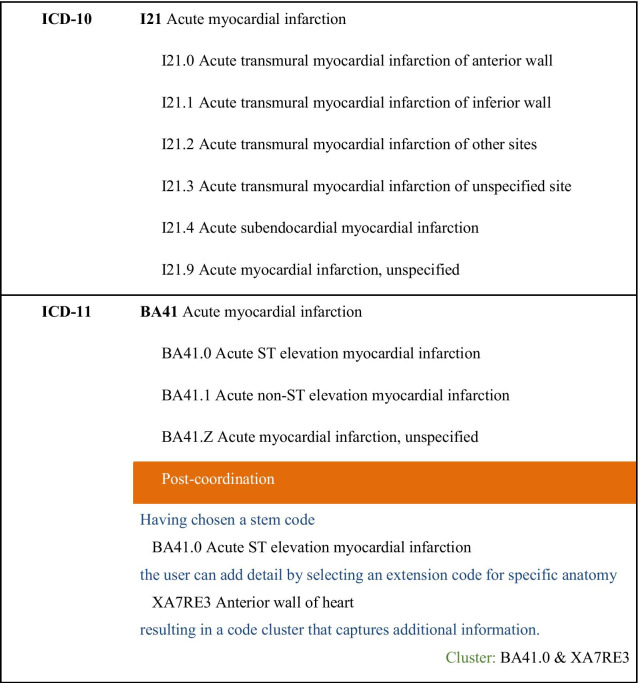
Acute myocardial infarction in ICD-10 and ICD-11-MMS

Acute myocardial infarction provides an example of how the 11th revision has affected coverage of an important cause of mortality and morbidity (Fig. 3). ICD-11-MMS provides a stem code with the same name and scope as that in ICD-10, to ensure that statistical time trends are comparable from ICD-10 to ICD-11-MMS. The WHO accepted advice that it is better to provide subcategories that are specified in terms of the presence of acute ST elevation than in terms of the affected part of the myocardium. It remains possible to code the part affected, but that is now done by adding an extension code to the code cluster representing the condition.

### Expected benefits of ICD-11-MMS

The new revision was designed to be capable of replacing all functions of ICD-10. In many contexts it will be capable of providing additional services or will provide existing functions in an improved way. This section describes what is expected in several areas.

#### Global reach, accessibility, and standardization

ICD-10 is used in many countries but little, if at all, in some others. The digital and web-based design of ICD-11-MMS will reduce certain barriers to wider use of the ICD [36]. The enhanced language support, the coding tool, the API on which it is based, and the applications that are expected to tap the potential of the API will make it easy for a person anywhere with web access to have essentially equivalent access to exactly the same version of ICD-11-MMS. Networks of ICD user agencies and individuals that exist in the era of ICD-10 are expected to develop further on the back of the inherently networked ICD-11-MMS.

#### Health metrics

For many decades, ICD-coded data have been the basis for international and national statistics on cause-specific mortality [37]. More recently, the mortality data, along with ICD-coded morbidity data, have been crucial inputs to estimates of burden of disease and injury at a global level [38] and more locally [39].

These measures commonly deal with fairly broad types of causes (e.g., tuberculosis, suicide), and ICD-11-MMS has been designed to provide statistical continuity for major causes as well as many specific causes. For example, the 264 causes [40] investigated in the Global Burden of Disease Study on the basis of data coded to ICD-10 and earlier revisions can be replicated using data coded to ICD-11-MMS. Mapping tables provided by the WHO will help to compile the same disease groups.

Some topics will be affected by the revision, particularly where category scope or placement have changed, or the basis for specifying subgroups. For example, statistics on the occurrence of transmural and subendothelial myocardial infarctions over time will be affected by the changes described in Fig. 3 unless all ICD-11-MMS data incorporate the anatomy extension code. Detailed analysis and bridge-coding studies [41] could assist in evaluating the impact of changes and provide a basis to allow for effects of the revision when interpreting statistics.

### Integrated support for hospital case data

The ICD has long been used to code records of hospital-admitted patients and some other types of clinical records. Many users found that the version of ICD-10 published by the WHO provided insufficient specificity for clinical purposes and for the related purpose of supporting activity-based billing and funding systems. Some WHO member countries developed “clinical modifications” of the ICD to serve these purposes. While similar in some respects, the various clinical modifications also differ considerably and do not provide an adequate basis for international comparisons [42].

ICD-11-MMS has been designed with these clinical purposes in mind, as well as mortality coding. Some parts of ICD-11-MMS draw on the clinical modifications of ICD-10 (e.g., the injury chapter), and the ICD-11 Foundation includes all categories that exist in the main clinical modifications of ICD-10. While WHO member states have yet to fully assess the sufficiency of ICD-11-MMS for these purposes, it is hoped that it will meet their requirements with much less need for modification than ICD-10 has, if any. Hence, ICD-11-MMS will facilitate international standardization of morbidity data in a way that ICD-10 could not.

#### Capability to operate within health information systems

The digital underpinning of ICD-11-MMS makes it a “native citizen” of the era of networked interoperating health information systems. Increasingly, data with different origins are combined for administrative or research purposes, enabling more value to be obtained from existing data. Where the sources combined include, for example, mortality and morbidity data, analysis and interpretation will be facilitated when the data in both sources are classified according to ICD-11-MMS.

As a classification designed with input from clinicians and agencies responsible for providing and administering health care, ICD-11-MMS is uniquely adapted to enable coherent and useful summarization of large volumes of disease data [43]. The data may originate in manual or electronic health records and be coded directly to ICD-11-MMS or via a structured terminology, such as SNOMED CT.

#### ICD-11-MMS support for activity-based funding systems

Activity-based systems, widely used to administer and allocate funds for hospital care, rely on coded diagnosis data [44, 45]. The similarities between ICD-10 and ICD-11-MMS and the great expressive potential of the new revision are reasons to expect that ICD-11-MMS will provide a good basis for activity-based systems. Rules on the extent of detail to be coded in a system may be needed to ensure that application of ICD-11-MMS results in minimal, if any, increase in coding burden, while tapping its flexibility and granularity. Once activity-based systems have migrated to ICD-11-MMS, greater international comparability may be achieved.

#### Quantitative derived measures

Case mix cost weights are long-established quantitative measures derived largely from ICD-coded data [46]. Other ICD-based quantitative derived measures have emerged, including measures of the probability of survival to discharge [47] and the presence of persisting disability after injury [48]. The great expressive power of ICD-11-MMS, particularly concerning aspects of case severity, is expected to support better performing quantitative measures on a wider range of topics.

### Governance, maintenance, and updates

We anticipate that preparations for implementation of ICD-11-MMS in a variety of settings will reveal some omissions and other potential for improvement that were not detected during pre-release testing. Naturally, new knowledge about diseases will also necessitate changes for as long as ICD-11-MMS is in use. The WHO has established two groups that will work together and with the WHO to maintain and update ICD-11: a Medical and Scientific Advisory Committee, comprised of medical and scientific experts, who validate the clinical and physiological basis as well as the ontological positioning of proposed entries into the Foundation, and a Classification and Statistics Advisory Committee, which will check proposals for their fitness for integration into the ICD-11-MMS classification and consider their possible impact on coding and coded data. The process for updating ICD-11 will be transparent and open. Anyone can lodge a proposal for updates through an online platform. The flexibility of the structure of ICD-11, combined with this updating mechanism, might obviate the need for another major revision for a considerable time.

### Next steps for countries

The adoption of ICD-11 in May 2019 marked the start of an implementation phase by WHO member states. In 2022, reporting of mortality data according to ICD-11-MMS will commence, with a transition period of at least five years. The nature, timing, and complexity of implementation for morbidity coding will vary from place to place. In general, the transition will be most complex in places in which ICD is used widely, in multiple systems, and underlies processes such as activity-based funding. The countries that are first to adopt ICD-11-MMS may well be some that do not have substantial legacy systems that will require alteration to accommodate the revision.

## Conclusions

Changes in content reflect developments in the understanding of diseases since ICD-10 was written. The ICD-11 rules for combining categories enable much better description of cases than has been possible previously, and its governance arrangements will ensure that it remains current. Information systems have changed more since ICD-10 was released than in the previous century. The era of globally networked and nearly real-time data systems has transformed many aspects of life, but health information has yet to fully make the transition. From its Foundation, information framework, and API to the suite of user-facing tools, ICD-11 has been designed to enable that transition to occur now. ICD-11 can be accessed at icd.who.int.

## Data Availability

ICD-11 can be accessed at icd.who.int.

## Abbreviations

API: Application programming interface
ICD: International classification of diseases
ICD-10: International classification of diseases, 10th revision
ICD-11: International classification of diseases, 11th revision
ICD-11-MMS: ICD-11 for mortality and morbidity statistics
URI: Unique resource identifier
WHO: World Health Organization

## References

[1] Wood PH (1990). Applications of the international classification of diseases. World Health Stat Q.

[2] World Health Organization. World health statistics. 2020. http://www.who.int/gho/publications/world_health_statistics/en. Accessed 12 Nov 2020

[3] World Health Organization. History of the development of the ICD. http://www.who.int/classifications/icd/en/HistoryOfICD.pdf. Accessed 12 Nov 2020.

[4] World Health Organization. International Classification of Diseases (ICD). https://www.who.int/standards/classifications/classification-of-diseases. Accessed 27 May 2021.

[5] White A (2014). Digital media and society: transforming economics, politics and social practices.

[6] World Health Organization. ICD-11 revision. https://icd.who.int/en. Accessed 12 Nov 2020.

[7] World Health Organization. ICD-11 Revision Conference. Tokyo Japan; 12–14 Oct 2016.

[8] Ghali WA, Pincus HA, Southern DA, Brien SE, Romano PS, Burnand B, et al. ICD-11 for quality and safety: overview of the WHO quality and safety Topic Advisory Group. Int J Qual Health Care. 2013;25:621–5. 10.1093/intqhc/mzt074 24154846

[9] World Health Organization. Groups that were involved in ICD-11 Revision Process. https://www.who.int/standards/classifications/classification-of-diseases/groups-that-were-involved-in-icd-11-revision-process. Accessed 28 May 2021.

[10] World Health Organization. ICD-11-MMS Joint Task Force (JTF). 2018. http://www.who.int/classifications/icd/revision/JTF_LOP.pdf. Accessed 12 Nov 2020.

[11] Reed GM, Roberts MC, Keeley J, Hooppell C, Matsumoto C, Sharan P (2013). Mental health professionals’ natural taxonomies of mental disorders: implications for the clinical utility of the ICD-11 and the DSM 5. J Clin Psychol.

[12] Roberts MC, Reed GM, Medina-Mora ME, Keeley JW, Sharan P, Johnson DK (2012). A global clinicians’ map of mental disorders to improve ICD-11: analysing meta-structure to enhance clinical utility. Int Rev Psychiatry.

[13] Stone J, Hallett M, Carson A, Bergen D, Shakir R (2014). Functional disorders in the neurology section of ICD-11: a landmark opportunity. Neurology.

[14] Rajakulendran S, Dua T, Harper M, Shakir R (2014). The classification of neurological disorders in the 11th revision of the International Classification of Diseases (ICD-11). J Neurol Neurosurg Psychiatry.

[15] Aymé S, Bellet B, Rath A (2015). Rare diseases in ICD11: making rare diseases visible in health information systems through appropriate coding. Orphanet J Rare Dis.

[16] Chute CG (2018). The rendering of human phenotype and rare diseases in ICD-11. J Inherit Metab Dis.

[17] Southern DA, Pincus HA, Romano PS, Bernard B, Harrison J, Forster AJ (2016). Enhanced capture of healthcare-related harms and injuries in the 11th revision of the International Classification of Diseases (ICD-11). Int J Qual Health Care.

[18] McKenzie K, Fingerhut L, Walker S, Harrison A, Harrison JE (2012). Classifying external causes of injury: history, current approaches, and future directions. Epidemiol Rev.

[19] Tanno LK, Calderon M, Linzer JF, Chalmers RJG, Demoly P (2017). Joint Allergy Academies. Collaboration between specialties for respiratory allergies in the International Classification of Diseases (ICD)-11. Respir Res.

[20] Tanno LK, Chalmers RJG, Calderon MA, Aymé S, Demoly P (2017). On behalf the Joint Allergy Academies. Reaching multidisciplinary consensus on classification of anaphylaxis for the eleventh revision of the World Health Organization’s (WHO) International Classification of Diseases (ICD-11). Orphanet J Rare Dis.

[21] World Health Organization. WHO Family of International Classifications (WHO-FIC) Network. 2018. http://www.who.int/classifications/network/en. Accessed 17 Nov 2020.

[22] World Health Organization. ICD11 Content Model Reference Guide. https://icd.who.int/icdapi/docs/ContentModelGuide.pdf. Accessed 28 May 2021.

[23] Shakir R, Davis S, Norrving B, Grisold W, Carroll WM, Feigin V, Hachinski V (2016). Revising the ICD: stroke is a brain disease. Lancet.

[24] Boerma T, Harrison J, Jakob R, Mathers C, Schmider A, Weber S (2016). Revising the ICD: explaining the WHO approach. Lancet.

[25] Rector A, Schulz S, Rodrigues JM, Chute CG, Solbrig H (2019). On beyond Gruber: “Ontologies” in today’s biomedical information systems and the limits of OWL. J Biomed Inform X.

[26] Rodrigues J-M, Schulz S, Rector A, Spackman K, Millar J, Campbell J (2014). ICD-11 and SNOMED CT common ontology: circulatory system. Stud Health Technol Inform.

[27] Schulz S, Rodrigues J-M, Rector A, Spackman K, Campbell J, Ustün B (2014). What’s in a class? Lessons learnt from the ICD—SNOMED CT harmonisation. Stud Health Technol Inform.

[28] SNOMED International. http://www.snomed.org/ Accessed 17 Nov 2020

[29] The Human Phenotype Ontology. https://hpo.jax.org/app/ Accessed 17 Nov 2020

[30] Harrison J, Mozzicato P (2009). MedDRA®: the tale of a terminology: side effects of drugs essay. Side Effects Drugs Annual.

[31] The Open Biological and Biomedical Ontology (OBO) Foundry. http://www.obofoundry.org/ Accessed 17 Nov 2020

[32] Cimino JJ (1998). Desiderata for controlled medical vocabularies in the twenty-first century. Methods Inf Med.

[33] World Health Organization. ICD APIs. https://icd.who.int/icdapi. Accessed 17 Nov 2020.

[34] World Health Organization. International Statistical Classification of Diseases and Related Health Problems—10th revision: Volume 2—instruction manual (2nd ed.). Geneva: World Health Organization; 2004.

[35] World Health Organization. ICD-11 coding tool. https://icd.who.int/ct11/icd11_mms/en/release. Accessed 12 Nov 2020.

[36] WHO. World Health Statistics 2020: Monitoring Health for the Sustainable Development Goals. Geneva, 2020. https://apps.who.int/iris/bitstream/handle/10665/332070/9789240005105-eng.pdf Accessed 25 Feb 2021.

[37] Lancet T (2017). Life, death, and disability in 2016. Lancet.

[38] Lancet T (2020). The Global Burden of Disease Study 2019. Lancet.

[39] Australian Institute of Health and Welfare. Australian Burden of Disease Study: impact and causes of illness and injury in Australia 2015. Australian Burden of Disease Study. Canberra: AIHW; 2019.

[40] GBD 2016 Causes of Death Collaborators (2017). Global, regional, and national age-sex specific mortality for 264 causes of death, 1980–2016: a systematic analysis for the Global Burden of Disease Study 2016. Lancet.

[41] Brocco S, Vercellino P, Goldoni C, Alba N, Gatti M (2010). «Bridge Coding» ICD-9, ICD-10 and effects on mortality statistics. Epidemiol Prev.

[42] Jetté N, Quan H, Hemmelgarn B, Drosler S, Maass C, Moskal L (2010). The development, evolution, and modifications of ICD-10: challenges to the international comparability of morbidity data. Med Care.

[43] Chen D, Zhang R, Zhao H, Feng J (2019). Bibliometric analysis of the development of ICD-11 in medical informatics. J Healthc Eng.

[44] O'Reilly J, Busse R, Häkkinen U, Or Z, Street A, Wiley M (2012). Paying for hospital care: the experience with implementing activity-based funding in five European countries. Health Econ Policy Law.

[45] Palmer KS, Agoritsas T, Martin D, Scott T, Mulla SM, Miller AP (2014). Activity-based funding of hospitals and its impact on mortality, readmission, discharge destination, severity of illness, and volume of care: a systematic review and meta-analysis. PLoS ONE.

[46] Pettengill J, Vertrees J (1982). Reliability and validity in hospital case-mix measurement. Health Care Financ Rev.

[47] Gagné M, Moore L, Beaudoin C, Batomen Kuimi BL, Sirois M-J (2016). Performance of International Classification of Diseases-based injury severity measures used to predict in-hospital mortality: a systematic review and meta-analysis. J Trauma Acute Care Surg.

[48] Gabbe BJ, Lyons RA, Simpson PM, Rivara FP, Ameratunga S, Polinder S (2016). Disability weights based on patient-reported data from a multinational injury cohort. Bull World Health Organ.

